# Supplementary material for: Cas13b-dependent and Cas13b-independent RNA knockdown of viral sequences in mosquito cells following guide RNA expression
Source: Commun Biol. 2020 Jul 31;3:413. doi: 10.1038/s42003-020-01142-6 (PMC7395101; doi:10.1038/s42003-020-01142-6)
Supplement: Supplementary file 1 — Supplementary Information [file 42003_2020_1142_MOESM1_ESM.pdf]

Supplementary Material

Supplementary Figures

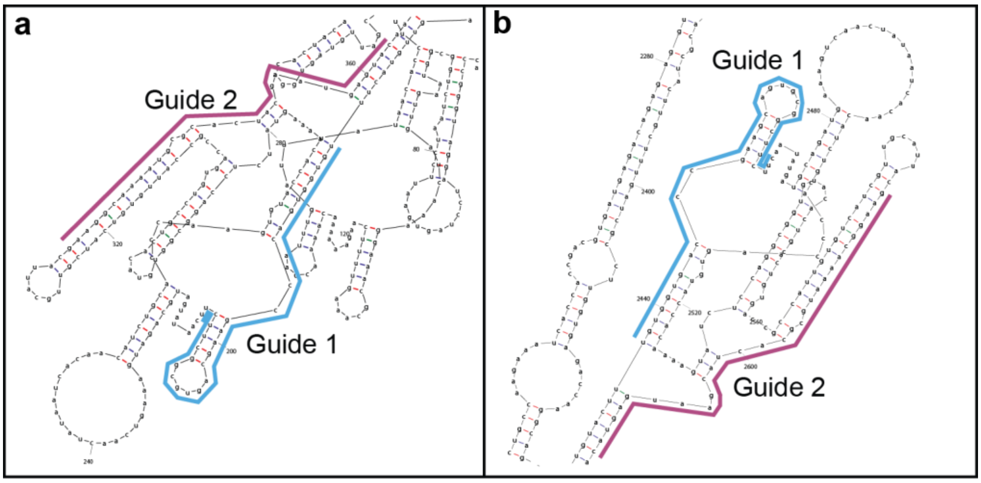

Supplementary Figure 1: Location of targets of guides 1 (blue) and 2 (purple) on the predicted RNA secondary structure (MFold [1]) of the chikungunya virus (CHIKV) nsP2 region of the (a) CHIKV-Luciferase reporter and (b) CHIKV. The two targets have similar predicted structures in the regions targeted by the guide RNAs.

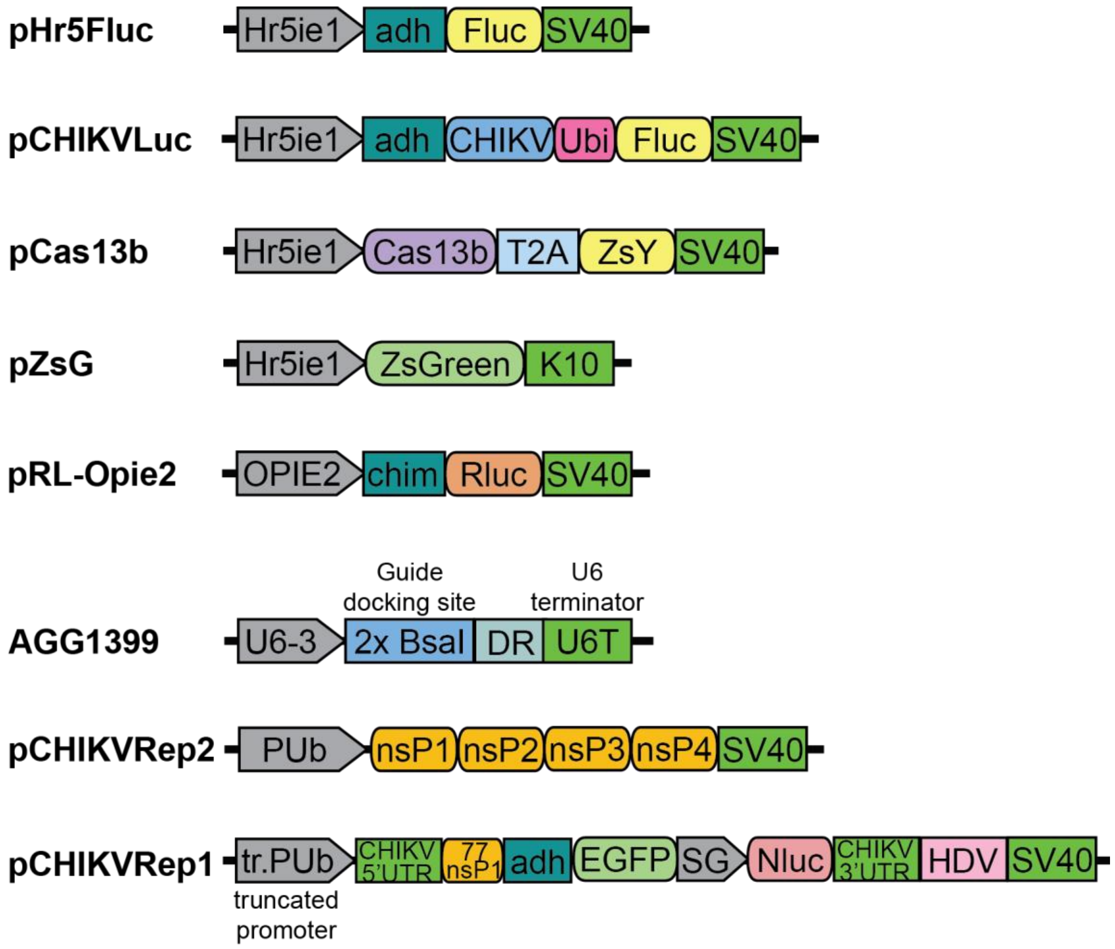

Supplementary Figure 2: Schematic of plasmids used in this study. Hr5ie1: baculovirus *Hr5-ie1* promoter, adh: *Drosophila melanogaster* alcohol dehydrogenase intron, Fluc: firefly luciferase, SV40: simian virus 40 polyadenylation signal, CHIKV: chikungunya virus sequence, Ubi: ubiquitin, Cas13b: PspCas13b of *Prevotella* sp. *P5-125* codon optimised for *Aedes aegypti*, T2A: thosea asigna virus 2A self-cleaving peptide, ZsY: ZsYellow fluorophore, ZsGreen: ZsGreen fluorophore, K10: 3' UTR of *D. melanogaster fs(1)K10* gene, OPIE2: *Orgyia pseudotsugata* multicapsid nucleopolyhedrovirus immediate early promoter, chim: chimeric intron, Rluc: *Renilla* luciferase, U6-3: *Ae. aegypti* U6-3 promoter, 2x BsaI: two *BsaI* restriction sites, DR: non-variable RNA guide backbone, U6T: U6 promoter terminator, PUb: *Ae. aegypti* polyubiquitin promoter, nsP1-4: CHIKV non-structural proteins 1-4, tr.PUb: truncated *Ae. aegypti* polyubiquitin promoter, UTR: untranslated region, 77 nsP1: 77nt of nsP1, EGFP: green fluorescent protein, SG: CHIKV subgenomic promoter, Nluc: nanoluciferase, HDV: Hepatitis delta virus ribozyme.

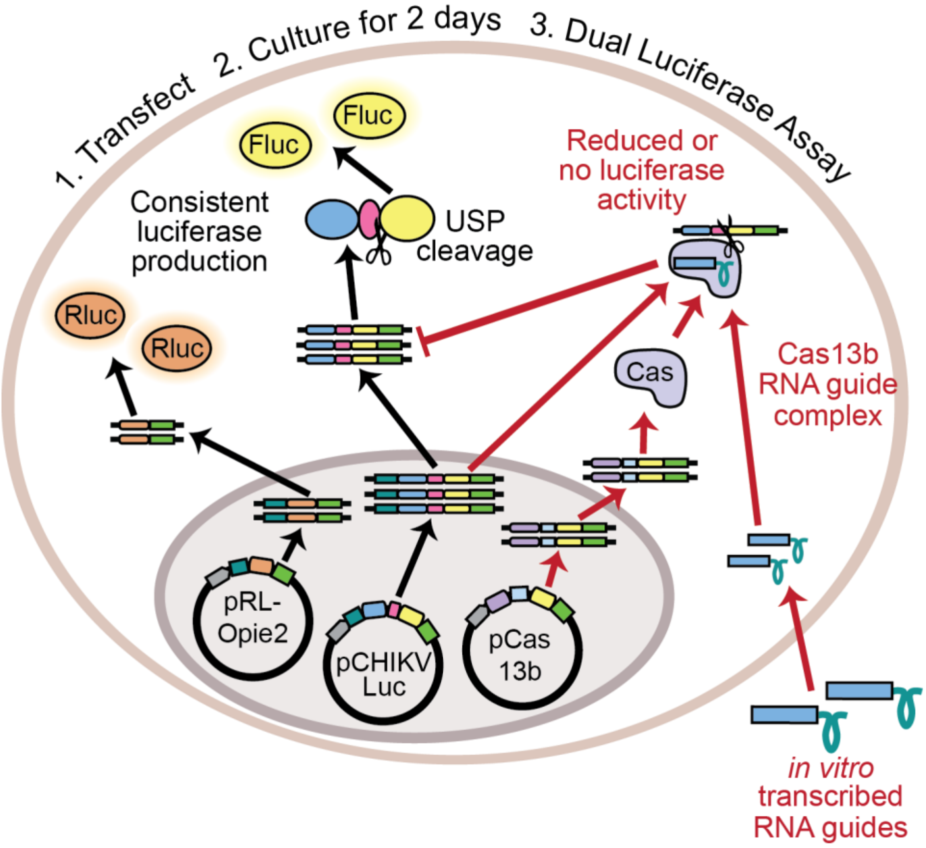

Supplementary Figure 3: Schematic of CHIKV-luciferase reporter assay with Cas13b (Cas) and *in vitro* transcribed RNA guides targeting chikungunya virus (CHIKV) nsP2 encoding region. Cas13b is expressed constitutively and the CHIKV-luciferase reporter produces a section of CHIKV nsP2 fused to firefly luciferase (Fluc) by ubiquitin. The targeting ability of the *in vitro* transcribed guides tested can be measured from the change in firefly luciferase expression levels. A *Renilla* luciferase (Rluc) plasmid (pRL-Opie2) is co-transfected as a control for transfection efficiency.

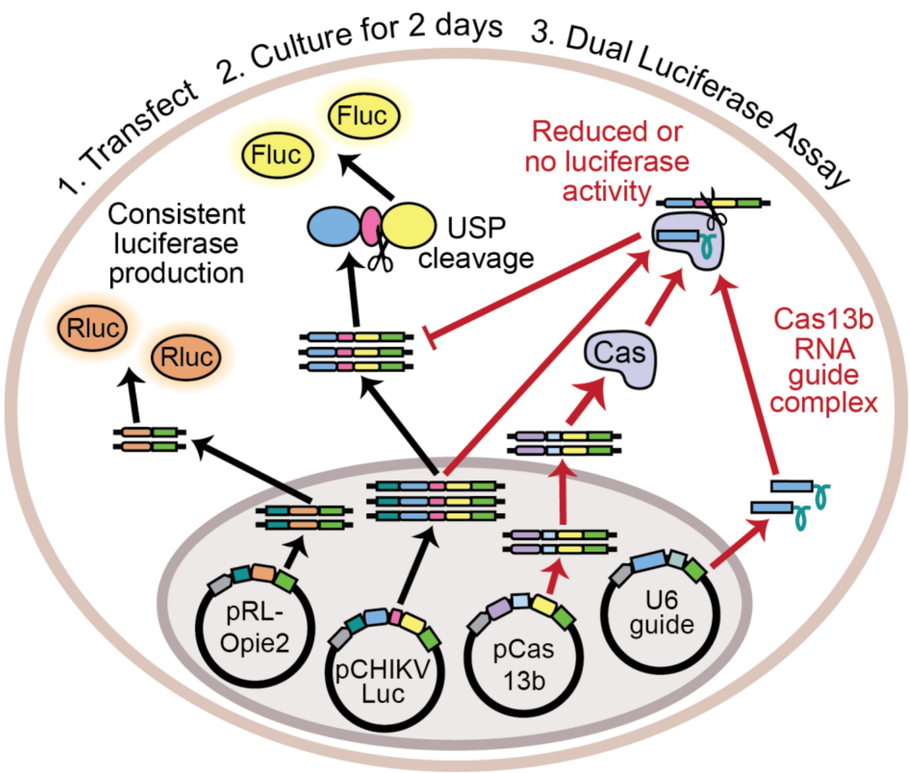

Supplementary Figure 4: Schematic of CHIKV-luciferase reporter assay with Cas13b (Cas) and Pol III promoter U6 driven RNA guides (U6 guide). Cas13b is expressed constitutively and the CHIKV-luciferase reporter produces a section of chikungunya virus (CHIKV) nsP2 fused to firefly luciferase (Fluc) by ubiquitin. The targeting ability of the U6 guides tested can be measured from the change in firefly luciferase expression levels. A *Renilla* luciferase (Rluc) plasmid (pRL-Opie2) is co-transfected as a control for transfection efficiency.

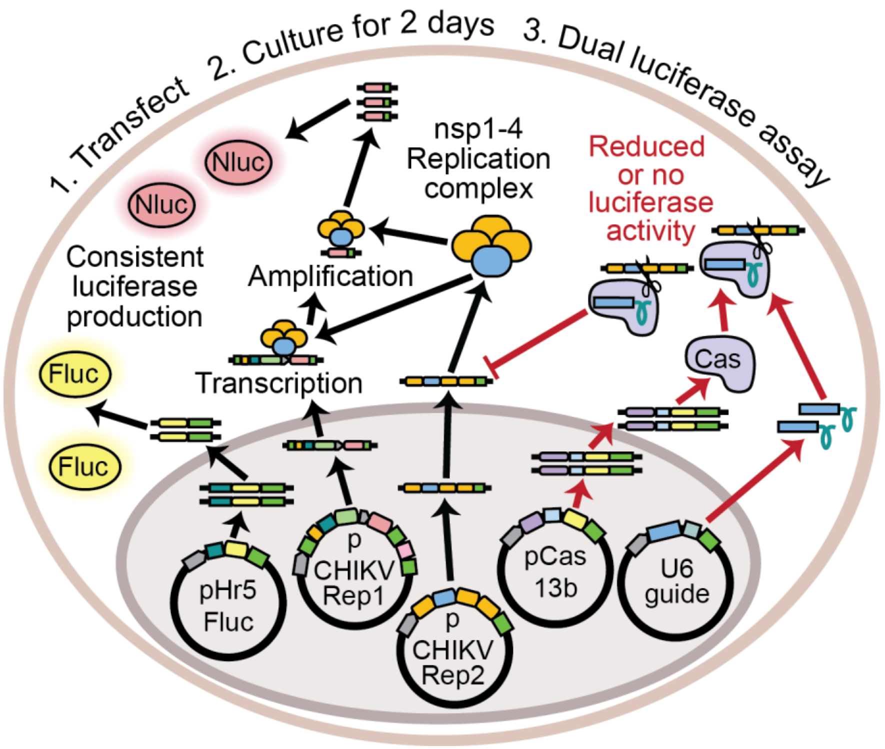

Supplementary Figure 5: Schematic of chikungunya virus (CHIKV) split replication assay with Cas13b (Cas) and Pol III promoter U6 promoter driven RNA guides (U6 guides). pCHIKVRep1 produces a modified CHIKV genome where the sequences encoding non-structural and structural proteins have been replaced with EGFP and nanoluciferase (Nluc), respectively. The viral replicase polyprotein is supplied in *trans* by the co-transfected pCHIKVRep2. The targeted nsP2 region encodes for a component of the CHIKV replication complex which is essential for the expression of Nluc that is under the control of the viral subgenomic promoter. The targeting ability of the U6 guides tested can be measured from the change in Nluc expression levels. A firefly luciferase plasmid (Fluc) is co-transfected as a control for transfection efficiency.

Supplementary Tables

Supplementary Table 1: Opening energies for guide target sites within RNA transcripts of reporters as predicted by RNAup [2]. Guide 1 target sites have higher energy requirements for opening than guide 2 target sites and are predicted to be less accessible.

Target site	Reporter	Predicted opening energy (kcal/mol)
1	CHIKV-luciferase	17.44
2	CHIKV-luciferase	10.26
1	Split replication system	16.07
2	Split replication system	12.14

Supplementary Table 2: Sequences of guides used (T7 or U6 promoter driven) as single guides or in the array. Lowercase: guide targeting region, uppercase: backbone, underlined text: string of 4 T's.

Type of guide	Sequence
Guide 1	ggaagaagccgcactgcttcgggtcaccacaGTTGTGGAAGGTCCAGTTTTGAGGGGCTATTACAAC
Guide 2	ggtactcattcgtagtgcgcattttgccttcGTTGTGGAAGGTCCAGTTTTGAGGGGCTATTACAAC
AmC1	ggcaccacggaggtgatgtgggccacggcgGTTGTGGAAGGTCCAGTTTTGAGGGGCTATTACAAC
AmC2	gtcccagccggtggtcttcttggccatcacgGTTGTGGAAGGTCCAGTTTTGAGGGGCTATTACAAC
AmC3	ggttcacgccgtggaaggtggacttgtgctcGTTGTGGAAGGTCCAGTTTTGAGGGGCTATTACAAC

Supplementary Table 3: Plasmid mixtures for each experiment.

In vitro transcribed single guides with synthetic reporter (Fig. 2 and Supplementary Fig. 3)	
Reporter	2ng pCHIKVLuc
Effector	20ng pCas13b or equimolar amount of pZsG
IV guides	10ng or 40ng of each *in vitro* transcribed guide (1, 2 or AmC3)
Transfection control	50ng pRL-Opie2
U6 single guides with synthetic reporter (Fig. 3 and Supplementary Fig. 4)	
Reporter	2ng pCHIKVLuc
Effector	20ng pCas13b or equimolar amount of pZsG
U6 guides	50ng of each U6-guide (1, 2 or AmC3)
Transfection control	50ng pRL-Opie2
U6 single guides with CHIKV split replication system (Fig. 4 and Supplementary Fig. 5)	
Reporter	10ng pCHIKVRep1, 1ng pCHIKVRep2
Effector	20ng pCas13b or equimolar amount of pZsG
U6 guides	50ng of each U6-guide (1, 2 or AmC3)
Transfection control	0.2ng pHr5Fluc
U6 array with synthetic reporter (Fig. 5)	
Reporter	2ng pCHIKVLuc
Effector	20ng pCas13b or equimolar amount of pZsG
IVT array	Each U6-array (U6-P1, U6-P2, U6-P3 or U6-PC) equimolar to 50ng U6-guide
Transfection control	50ng pRL-Opie2

Supplementary Table 4: Results of statistical analyses performed for transfections with *in vitro* transcribed guides and CHIKV luciferase in Aag2 cells.

Aag2	IVT guides	40ng			
Differences were based on squareroot transformed data.					
**lme model**	**Estimate**	**Std. Error**	**df**	**t value**	**Pr(>|t|)**
Presence of Cas13b	0.068	0.06	102.00004	1.1	0.274
Guide 1	-0.858	0.07	102.00004	-11.4	< 2e-16
Guide 2	-3.59	0.07	102.00004	-47.7	< 2e-16
Interaction Cas13b:Guide					0.288
**Post-hoc Analysis**	**Estimate**	**Std. Error**	**z value**	**Pr(>|z|)**	
1 - C	-0.858	0.07	-11.4	<2e-16	
2 - C	-3.59	0.07	-47.7	<2e-16	
2--1	-2.731	0.07	-36.4	<2e-16	
**Random Effects**	**Variance**	**Std. Dev.**			
Experiment	0.01483	0.1218			
Residuals	0.10184	0.3191			

Aag2	IVT guides	10ng			
Differences were based on log2 transformed data.					
**lme model**	**Estimate**	**Std. Error**	**df**	**t value**	**Pr(>|t|)**
Presence of Cas13b	0.008	0.07	102.00208	0.105	0.917
Guide 1	-0.761	0.08	102.00208	-8.86	2.74E-14
Guide 2	-4.003	0.08	102.00208	-46.6	< 2e-16
Interaction Cas13b:Guide					0.956
**Post-hoc Analysis**	**Estimate**	**Std. Error**	**z value**	**Pr(>|z|)**	
1 - C	-0.761	0.08	-8.86	<2e-16	
2 - C	-4.003	0.08	-46.6	<2e-16	
2--1	-3.242	0.08	-37.8	<2e-16	
**Random Effects**	**Variance**	**Std. Dev.**			
Experiment	0.001171	0.03422			
Residuals	0.132878	0.36452			

Supplementary Table 5: Results of statistical analyses performed for transfections with *in vitro* transcribed guides and CHIKV luciferase in AF05 cells.

AF05 IVT guides 40ng

Differences were based on log2 transformed data.

lme model	Estimate	Std. Error	df	t value	Pr(>|t|)
Presence of Cas13b	0.341	0.09	100	3.47	0.0008
Guide 1	-0.939	0.09	100	-9.56	9.27E-16
Guide 2	-6.67	0.09	100	-67.92	< 2e-16
Interaction Cas13b:Guide					0.0435
Interaction Cas13b-Guide1	0.038	0.13	100	0.28	0.786
Interaction Cas13b-Guide2	-0.286	0.13	100	-2.054	0.0426

Post-hoc Analysis	Estimate	Std. Error	z value	Pr(>|z|)
Cas13 Guide 1 - Cas13 Guide C	-0.939	0.09	-9.557	< 0.001
Cas13 Guide 2 - Cas13 Guide C	-6.67	0.09	-67.911	< 0.001
ZsG Guide C - Cas13 Guide C	0.341	0.09	3.468	0.00695
Cas13 Guide 2 - Cas13 Guide 1	-5.74	0.09	-58.354	< 0.001
ZsG Guide 1 - Cas13 Guide 1	0.379	0.09	3.854	0.00168
ZsG Guide 2 - Cas13 Guide 2	0.056	0.09	0.563	0.993
ZsG Guide 1 - ZsG Guide C	-0.901	0.09	-9.171	< 0.001
ZsG Guide 2 - ZsG Guide C	-6.96	0.09	-70.815	< 0.001
ZsG Guide 2 - ZsG Guide 1	-6.06	0.09	-61.644	< 0.001

Random Effects	Variance	Std. Dev.
Experiment	0.01365	0.1168
Residuals	0.0868	0.2946

AF05 IVT guides 10ng

Differences were based on untransformed data.

lme model	Estimate	Std. Error	df	t value	Pr(>|t|)
Presence of Cas13b	-1.15	0.88	102	-1.29	0.2001
Guide 1	-11.9	1.08	102	-10.93	< 2e-16
Guide 2	-29.4	1.08	102	-26.99	< 2e-16
Interaction Cas13b:Guide					0.161

Post-hoc Analysis	Estimate	Std. Error	z value	Pr(>|z|)
1 - C	-11.9	1.08	-10.9	<2e-16
2 - C	-29.4	1.08	-26.99	<2e-16
2--1	-17.5	1.08	-16.05	<2e-16

Random Effects	Variance	Std. Dev.
Experiment	1.653	1.286
Residuals	21.353	4.621

Supplementary Table 6*:* Results of statistical analyses performed for transfections with *in vitro* transcribed guides and CHIKV luciferase in AF319 cells.

AF319 IVT guides 40ng

Differences were based on log2 transformed data.

lme model	Estimate	Std. Error	df	t value	Pr(>|t|)
Presence of Cas13b	0.352	0.11	99.999997	3.02	0.00317
Guide 1	-0.557	0.11	99.999997	-4.78	5.98E-06
Guide 2	-4.21	0.11	99.999997	-36.14	< 2e-16
Interaction Cas13b-Guide1	-0.006	0.16	99.999997	-0.034	0.973
Interaction Cas13b-Guide2	0.801	0.16	99.999997	4.87	4.23E-06
**Post-hoc Analysis**	**Estimate**	**Std. Error**	**z value**	**Pr(>|z|)**	
Cas13 Guide 1 - Cas13 Guide C	-0.557	0.11	-4.79	<0.001	
Cas13 Guide 2 - Cas13 Guide C	-4.21	0.11	-36.1	<0.001	
ZsG Guide C - Cas13 Guide C	0.352	0.11	3.02	0.0301	
Cas13 Guide 2 - Cas13 Guide 1	-3.65	0.11	-31.4	<0.001	
ZsG Guide 1 - Cas13 Guide 1	0.347	0.11	2.98	0.0348	
ZsG Guide 2 - Cas13 Guide 2	1.16	0.11	9.91	<0.001	
ZsG Guide 1 - ZsG Guide C	-0.562	0.11	-4.83	<0.001	
ZsG Guide 2 - ZsG Guide C	-3.41	0.11	-29.3	<0.001	
ZsG Guide 2 - ZsG Guide 1	-2.85	0.11	-24.4	<0.001	
**Random Effects**	**Variance**	**Std. Dev.**			
Experiment	0.1566	0.3957			
Residuals	0.1219	0.3491			

AF319 IVT guides 10ng

Differences were based on log2 transformed data.

lme model	Estimate	Std. Error	df	t value	Pr(>|t|)
Presence of Cas13b	0.291	0.05	102	5.78	8.33E-08
Guide 1	-0.304	0.06	102	-4.93	3.31E-06
Guide 2	-0.936	0.06	102	-15.2	< 2e-16
Interaction Cas13:Guide					0.761
**Post-hoc Analysis**	**Estimate**	**Std. Error**	**z value**	**Pr(>|z|)**	
1 - C	-0.304	0.06	-4.92	2.59E-06	
2 - C	-0.936	0.06	-15.2	< 1e-06	
2--1	-0.633	0.06	-10.3	< 1e-06	
**Random Effects**	**Variance**	**Std. Dev.**			
Experiment	0.07086	0.2662			
Residuals	0.06825	0.2613			

Supplementary Table 7: Results of statistical analyses performed for transfections with *in vitro* transcribed guides and CHIKV luciferase in C6/36 cells.

C6/36 IVT guides 40ng

Differences were based on untransformed data.

2 way ANOVA	Df	Sum Sq	Mean Sq	F value	Pr(>F)
Guide	2	0.237	0.11849	34.348	3.99E-06
Presence of Cas13b	1	0.00177	0.00177	0.512	0.486
Residuals	14	0.0483	0.00345		
Interaction Cas13:Guide	2	0.00046	0.00023	0.058	0.944

Post-hoc Analysis	Estimate	Std. Error	t-value	Pr(>|t|)
1--2	0.244	0.03391	7.197	<1e-04
C - 2	0.243	0.03391	7.16	<1e-04
C - 1	-0.00131	0.03391	-0.039	0.999

C6/36 IVT guides 10ng

Differences were based on squareroot transformed data.

lme model	Estimate	Std. Error	df	t value	Pr(>|t|)
Presence of Cas13b	-0.114	0.01	102	-7.91	3.21E-12
Guide 1	-0.042	0.01	102	-2.36	0.0207
Guide 2	-0.153	0.01	102	-8.67	7.34E-14
Interaction Cas13:Guide					0.6704

Post-hoc Analysis	Estimate	Std. Error	z value	Pr(>|z|)
1 - C	-0.042	0.01	-2.36	0.0491
2 - C	-0.153	0.01	-8.67	<0.001
2--1	-0.111	0.01	-6.32	<0.001

Random Effects	Variance	Std. Dev.
Experiment	0.002993	0.05471
Residuals	0.005566	0.07461

Supplementary Table 8: Results of statistical analyses performed for transfections with U6 guides and CHIKV luciferase in Aag2 cells.

Aag2	U6 guides				
Differences were based on untransformed data.					
lme model	Estimate	Std. Error	df	t value	Pr(>|t|)
Presence of Cas13b	-6.79	1.31	111.035	-5.16	0.0000011
Guide 1	-13.1	1.29	111.025	-10.03	< 2e-16
Guide 2	-14.5	1.29	111.025	-11.13	< 2e-16
Interaction Cas13b-Guide1	12.1	1.84	111.03	6.51	2.26E-09
Interaction Cas13b-Guide2	11.6	1.84	111.03	6.24	8.27E-09
Post-hoc Analysis	Estimate	Std. Error	z value	Pr(>|z|)	
Cas13 Guide 1 - Cas13 Guide C	-13.1	1.29	-10.03	<0.001	
Cas13 Guide 2 - Cas13 Guide C	-14.5	1.29	-11.13	<0.001	
ZsG Guide C - Cas13 Guide C	-6.79	1.31	-5.16	<0.001	
Cas13 Guide 2 - Cas13 Guide 1	-1.43	1.29	-1.1	0.883	
ZsG Guide 1 - Cas13 Guide 1	5.25	1.29	4.04	<0.001	
ZsG Guide 2 - Cas13 Guide 2	4.75	1.29	3.66	0.00358	
ZsG Guide 1 - ZsG Guide C	-0.991	1.31	-0.76	0.975	
ZsG Guide 2 - ZsG Guide C	-2.92	1.31	-2.22	0.2295	
ZsG Guide 2 - ZsG Guide 1	-1.93	1.29	-1.49	0.674	
Random Effects	Variance	Std. Dev.			
Experiment	5.103	2.259			
Residuals	16.865	4.107			

Supplementary Table 9: Results of statistical analyses performed for transfections with U6 guides and CHIKV luciferase in AF05 cells.

AF05	U6 guides				
Differences were based on untransformed data.					
lme model	Estimate	Std. Error	df	t value	Pr(>|t|)
Presence of Cas13b	-5.26	1	100	-5.26	8.51E-07
Guide 1	-18.9	1	100	-18.9	<2.00E-16
Guide 2	-22.4	1	100	-22.35	<2.00E-16
Interaction Cas13b-Guide1	14.91	1.41	100	10.54	<2.00E-16
Interaction Cas13b-Guide2	14.2	1.41	100	10.03	<2.00E-16
Interaction Cas13:Guide	1271	635	100	70.59	<2.00E-16
Post-hoc Analysis	Estimate	Std. Error	z value	Pr(>|z|)	
Cas13 Guide 1 - Cas13 Guide C	-18.9	1	-18.9	<0.001	
Cas13 Guide 2 - Cas13 Guide C	-22.4	1	-22.35	<0.001	
ZsG Guide C - Cas13 Guide C	-5.26	1	-5.26	<0.001	
Cas13 Guide 2 - Cas13 Guide 1	-3.46	1	-3.46	0.00715	
ZsG Guide 1 - Cas13 Guide 1	9.65	1	9.65	<0.001	
ZsG Guide 2 - Cas13 Guide 2	8.93	1	8.93	<0.001	
ZsG Guide 1 - ZsG Guide C	-3.993	1	-3.993	<0.001	
ZsG Guide 2 - ZsG Guide C	-8.18	1	-8.18	<0.001	
ZsG Guide 2 - ZsG Guide 1	-4.18	1	-4.18	<0.001	
Random Effects	Variance	Std. Dev.			
Experiment	1.137	1.066			
Residuals	9.003	3			

Supplementary Table 10: Results of statistical analyses performed for transfections with U6 guides and CHIKV luciferase in AF319 cells.

<table>
<tr><td>AF319</td><td>U6 guides</td><td></td><td></td><td></td><td></td></tr>
<tr><td colspan="6">Differences were based on log2 transformed data.</td></tr>
<tr><th>lme model</th><th>Estimate</th><th>Std. Error</th><th>df</th><th>t value</th><th>Pr(>|t|)</th></tr>
<tr><td>Interaction Cas13:Guide</td><td></td><td></td><td></td><td>4.5268</td><td>0.01312</td></tr>
<tr><td>Presence of Cas13b</td><td>0.723</td><td>0.14</td><td>100</td><td>5.13</td><td>1.44E-06</td></tr>
<tr><td>Guide 1</td><td>-1.19</td><td>0.14</td><td>100</td><td>-8.38</td><td>3.45E-13</td></tr>
<tr><td>Guide 2</td><td>-1.94</td><td>0.14</td><td>100</td><td>-13.8</td><td><2e-16</td></tr>
<tr><td>Interaction Cas13b-Guide1</td><td>0.551</td><td>0.199</td><td>100</td><td>2.77</td><td>0.00682</td></tr>
<tr><td>Interaction Cas13b-Guide2</td><td>0.481</td><td>0.199</td><td>100</td><td>2.42</td><td>0.01762</td></tr>
<tr><th>Post-hoc Analysis</th><th>Estimate</th><th>Std. Error</th><th>z value</th><th>Pr(>|z|)</th><th></th></tr>
<tr><td>Cas13 Guide 1 - Cas13 Guide C</td><td>-1.181</td><td>0.14</td><td>-8.38</td><td><0.001</td><td></td></tr>
<tr><td>Cas13 Guide 2 - Cas13 Guide C</td><td>-1.935</td><td>0.14</td><td>-13.8</td><td><0.001</td><td></td></tr>
<tr><td>ZsG Guide C - Cas13 Guide C</td><td>0.723</td><td>0.14</td><td>5.13</td><td><0.001</td><td></td></tr>
<tr><td>Cas13 Guide 2 - Cas13 Guide 1</td><td>-0.755</td><td>0.14</td><td>-5.36</td><td><0.001</td><td></td></tr>
<tr><td>ZsG Guide 1 - Cas13 Guide 1</td><td>1.273</td><td>0.14</td><td>9.04</td><td><0.001</td><td></td></tr>
<tr><td>ZsG Guide 2 - Cas13 Guide 2</td><td>1.204</td><td>0.14</td><td>8.54</td><td><0.001</td><td></td></tr>
<tr><td>ZsG Guide 1 - ZsG Guide C</td><td>-0.631</td><td>0.14</td><td>-4.48</td><td><0.001</td><td></td></tr>
<tr><td>ZsG Guide 2 - ZsG Guide C</td><td>-1.454</td><td>0.14</td><td>-10.4</td><td><0.001</td><td></td></tr>
<tr><td>ZsG Guide 2 - ZsG Guide 1</td><td>-0.824</td><td>0.14</td><td>-5.85</td><td><0.001</td><td></td></tr>
<tr><th>Random Effects</th><th>Variance</th><th>Std. Dev.</th><th></th><th></th><th></th></tr>
<tr><td>Experiment</td><td>0.007618</td><td>0.08728</td><td></td><td></td><td></td></tr>
<tr><td>Residuals</td><td>0.178612</td><td>0.42262</td><td></td><td></td><td></td></tr>
</table>

Supplementary Table 11: Results of statistical analyses performed for transfections with U6 guides and CHIKV luciferase in C6/36 cells.

C6/36	U6 guides				
Differences were based on log2 transformed data.					
lme model	Estimate	Std. Error	df	t value	Pr(>|t|)
Presence of Cas13b	-0.323	0.13	100	-2.31	0.0231
Guide 1	-2.38	0.13	100	-16.96	<2.00E-16
Guide 2	-2.6	0.13	100	-18.6	<2.00E-16
Interaction Cas13b-Guide1	1.6	0.19	100	8.06	1.69E-12
Interaction Cas13b-Guide2	1.35	0.19	100	6.82	7.26E-10
Interaction Cas13:Guide	13.3	6.61	100	37.7	6.47E-13
Post-hoc Analysis	Estimate	Std. Error	z value	Pr(>|z|)	
Cas13 Guide 1 - Cas13 Guide C	-2.38	0.13	-16.96	<0.001	
Cas13 Guide 2 - Cas13 Guide C	-2.6	0.13	-18.6	<0.001	
ZsG Guide C - Cas13 Guide C	-0.33	0.13	-2.31	0.191	
Cas13 Guide 2 - Cas13 Guide 1	-0.23	0.13	-1.6	0.602	
ZsG Guide 1 - Cas13 Guide 1	1.28	0.13	9.1	<0.001	
ZsG Guide 2 - Cas13 Guide 2	1.03	0.13	7.33	<0.001	
ZsG Guide 1 - ZsG Guide C	-0.78	0.13	-5.57	<0.001	
ZsG Guide 2 - ZsG Guide C	-1.25	0.13	-8.92	<0.001	
ZsG Guide 2 - ZsG Guide 1	-0.47	0.13	-3.36	0.0102	
Random Effects	Variance	Std. Dev.			
Experiment	0.386	0.6213			
Residuals	0.1758	0.4192			

Supplementary Table 12: Results of statistical analyses performed for transfections with U6 guides and CHIKV split replication system in Aag2 cells.

Aag2 Split-replication system	U6 guide					
Differences based on untransformed data.						
**lme model**	**Sum Sq**	**Mean Sq**	**NumDF**	**DenDF**	**F value**	**Pr(>F)**
Presence of Cas13b	270620	270620	1	100	60.406	7.00E-12
Guide	3268688	1634344	2	100	364.808	< 2.2e-16
Interaction Cas13b:Guide	257345	128672	2	100	28.721	1.39E-10

	Estimate	Std. Error	df	t value	Pr(>|t|)
(Intercept)	637.333	21.433	6.559	29.736	3.04E-08
Presence of Cas13b	-35.222	22.311	100	-1.579	0.118
Guide 1	-281.889	22.311	100	-12.635	< 2e-16
Guide 2	-512.9	22.311	100	-22.989	< 2e-16
Interaction Cas13 - Guide 1	226.667	31.552	100	7.184	1.23E-10
Interaction Cas13 - Guide 2	179.344	31.552	100	5.684	1.30E-07

Post-hoc Analysis	Estimate	Std. Error	z value	Pr(>|z|)
Cas13 Guide 1 - Cas13 Guide C	-281.89	22.31	-12.635	< 0.001
Cas13 Guide 2 - Cas13 Guide C	-512.9	22.31	-22.989	< 0.001
ZsG Guide C - Cas13 Guide C	-35.22	22.31	-1.579	0.61271
Cas13 Guide 2 - Cas13 Guide 1	-231.01	22.31	-10.354	< 0.001
ZsG Guide 1 - Cas13 Guide 1	191.44	22.31	8.581	< 0.001
ZsG Guide 2 - Cas13 Guide 2	144.12	22.31	6.46	< 0.001
ZsG Guide 1 - ZsG Guide C	-55.22	22.31	-2.475	0.13147
ZsG Guide 2 - ZsG Guide C	-333.56	22.31	-14.95	< 0.001
ZsG Guide 2 - ZsG Guide 1	-278.33	22.31	-12.475	< 0.001

Random Effects	Variance	Std. Dev.
Experiment	631.4	25.13
Residuals	4480	66.93

Supplementary Table 13: Results of statistical analyses performed for transfections with U6 guides and CHIKV split replication system in AF05 cells.

AF05 Split-replication system **U6 guide**

Differences based on untransformed data.

lme model	Sum Sq	Mean Sq	NumDF	DenDF	F value	Pr(>F)
Presence of Cas13b	1714356	1714356	1	100	267.52	< 2.2e-16
Guide	4252639	2126320	2	100	331.81	< 2.2e-16
Interaction Cas13b:Guide	401545	200773	2	100	31.33	2.73E-11

	Estimate	Std. Error	df	t value	Pr(>|t|)
(Intercept)	778.333	32.674	3.824	23.822	2.67E-05
Presence of Cas13b	85.5	26.684	100	3.204	0.00182
Guide 1	-226.222	26.684	100	-8.478	2.11E-13
Guide 2	-561.222	26.684	100	-21.032	< 2e-16
Interaction Cas13 - Guide 1	288.722	37.737	100	7.651	1.27E-11
Interaction Cas13 - Guide 2	210.722	37.737	100	5.584	2.03E-07

Post-hoc Analysis	Estimate	Std. Error	z value	Pr(>|z|)
Cas13 Guide 1 - Cas13 Guide C	-226.22	26.68	-8.478	<0.001
Cas13 Guide 2 - Cas13 Guide C	-561.22	26.68	-21.032	<0.001
ZsG Guide C - Cas13 Guide C	85.5	26.68	3.204	0.0171
Cas13 Guide 2 - Cas13 Guide 1	-335	26.68	-12.554	<0.001
ZsG Guide 1 - Cas13 Guide 1	374.22	26.68	14.024	<0.001
ZsG Guide 2 - Cas13 Guide 2	296.22	26.68	11.101	<0.001
ZsG Guide 1 - ZsG Guide C	62.5	26.68	2.342	0.1773
ZsG Guide 2 - ZsG Guide C	-350.5	26.68	-13.135	<0.001
ZsG Guide 2 - ZsG Guide 1	-413	26.68	-15.477	<0.001

Random Effects	Variance	Std. Dev.
Experiment	2135	46.2
Residuals	6408	80.05

Supplementary Table 14: Results of statistical analyses performed for transfections with U6 guides and CHIKV split replication system in AF319 cells.

AF319 Split-replication system **U6 guide**

Differences based on squareroot transformed data.

2-way ANOVA	Df	Sum Sq	Mean Sq	F value	Pr(>F)
Guide	2	362.5	181.27	420.369	7.77E-12
Presence of Cas13b	1	4.2	4.22	9.779	0.00874
Interaction Cas13b:Guide	2	59.8	29.91	69.366	2.55E-07
Residuals	12	5.2	0.43		

Post-hoc Analysis	Estimate	Std. Error	t value	Pr(>|t|)
Cas13 Guide 1 - Cas13 Guide 2	7.3355	0.5362	13.682	< 0.001
Cas13 Guide C - Cas13 Guide 2	15.1433	0.5362	28.244	< 0.001
ZsG Guide 2 - Cas13 Guide 2	4.3344	0.5362	8.084	< 0.001
Cas13 Guide C - Cas13 Guide 1	7.8078	0.5362	14.562	< 0.001
ZsG Guide 1 - Cas13 Guide 1	2.6673	0.5362	4.975	0.0033
ZsG Guide C - Cas13 Guide C	-4.0977	0.5362	-7.643	< 0.001
ZsG Guide 1 - ZsG Guide 2	5.6685	0.5362	10.572	< 0.001
ZsG Guide C - ZsG Guide 2	6.7113	0.5362	12.517	< 0.001
ZsG Guide C - ZsG Guide 1	1.0428	0.5362	1.945	0.42311

Supplementary Table 15: Results of statistical analyses performed for transfections with U6 guides and CHIKV split replication system in C6/36 cells.

C6/36 Split-replication system **U6 guide**

Differences based on untransformed data.

lme model	Sum Sq	Mean Sq	NumDF	DenDF	F value	Pr(>F)
Presence of Cas13b	83222	83222	1	100	6.8854	0.01005
Guide	2443823	1221912	2	100	101.0956	< 2.2e-16
Interaction Cas13b:Guide	572400	286200	2	100	23.6789	3.81E-09

	Estimate	Std. Error	df	t value	Pr(>|t|)
(Intercept)	870.944	38.797	5.032	22.449	3.07E-06
Presence of Cas13b	-150.333	36.646	100	-4.102	8.35E-05
Guide 1	-373.167	36.646	100	-10.183	< 2e-16
Guide 2	-518.667	36.646	100	-14.153	< 2e-16
Interaction Cas13 - Guide 1	313.111	51.826	100	6.042	2.63E-08
Interaction Cas13 - Guide 2	304.444	51.826	100	5.874	5.59E-08

Post-hoc Analysis	Estimate	Std. Error	z value	Pr(>|z|)
Cas13 Guide 1 - Cas13 Guide C	-373.167	36.646	-10.183	< 1e-04
Cas13 Guide 2 - Cas13 Guide C	-518.667	36.646	-14.153	< 1e-04
ZsG Guide C - Cas13 Guide C	-150.333	36.646	-4.102	0.000566
Cas13 Guide 2 - Cas13 Guide 1	-145.5	36.646	-3.97	0.001061
ZsG Guide 1 - Cas13 Guide 1	162.778	36.646	4.442	0.000126
ZsG Guide 2 - Cas13 Guide 2	154.111	36.646	4.205	0.000353
ZsG Guide 1 - ZsG Guide C	-60.056	36.646	-1.639	0.572644
ZsG Guide 2 - ZsG Guide C	-214.222	36.646	-5.846	< 1e-04
ZsG Guide 2 - ZsG Guide 1	-154.167	36.646	-4.207	0.000373

Random Effects	Variance	Std. Dev.
Experiment	2501	50.01
Residuals	12087	109.94

Supplementary Table 16: Results of statistical analyses performed for transfections with U6 array and CHIKV luciferase in Aag2 cells.

Aag2	U6 array					
Differences were based on untransformed data.						
lme model	Sum Sq	Mean Sq	NumDF	DenDF	F value	Pr(>F)
Presence of Cas13b	501.6	501.59	1	134	52.669	2.88E-11
Position in array	6835.1	2278.36	3	134	239.236	< 2.2e-16
Interaction Cas13:Position	1216.8	405.6	3	134	42.59	< 2.2e-16
	Estimate	Std. Error	df	t value	Pr(>|t|)	
(Intercept)	25.8	1.013	6.575	25.471	8.09E-08	
Presence of Cas13b	-4.446	1.029	134	-4.322	3.00E-05	
PositionP1	-23.624	1.029	134	-22.965	< 2e-16	
PositionP2	-21.727	1.029	134	-21.122	< 2e-16	
PositionP3	-18.674	1.029	134	-18.154	< 2e-16	
Interaction of Cas13 - PositionP1	15.878	1.455	134	10.914	< 2e-16	
Interaction of Cas13 - PositionP2	10.53	1.455	134	7.238	3.18E-11	
Interaction of Cas13 - PositionP3	6.306	1.455	134	4.335	2.85E-05	
Post-hoc Analysis	Estimate	Std. Error	z value	Pr(>|z|)		
Cas13.P1 - Cas13.C	-23.624	1.029	-22.965	<0.001		
Cas13.P2 - Cas13.C	-21.727	1.029	-21.122	<0.001		
Cas13.P3 - Cas13.C	-18.674	1.029	-18.154	<0.001		
ZsG.C - Cas13.C	-4.446	1.029	-4.322	<0.001		
Cas13.P2 - Cas13.P1	1.896	1.029	1.844	0.5901		
Cas13.P3 - Cas13.P1	4.95	1.029	4.812	<0.001		
ZsG.P1 - Cas13.P1	11.432	1.029	11.113	<0.001		
Cas13.P3 - Cas13.P2	3.053	1.029	2.968	0.0603		
ZsG.P2 - Cas13.P2	6.084	1.029	5.915	<0.001		
ZsG.P3 - Cas13.P3	1.86	1.029	1.808	0.6145		
ZsG.P1 - ZsG.C	-7.746	1.029	-7.53	<0.001		
ZsG.P2 - ZsG.C	-11.198	1.029	-10.885	<0.001		
ZsG.P3 - ZsG.C	-12.368	1.029	-12.024	<0.001		
ZsG.P2 - ZsG.P1	-3.451	1.029	-3.355	0.0181		
ZsG.P3 - ZsG.P1	-4.622	1.029	-4.494	<0.001		
ZsG.P3 - ZsG.P2	-1.171	1.029	-1.138	0.9484		
Random Effects	Variance	Std. Dev.				
Experiment	1.491	1.221				
Residuals	9.523	3.086				

Supplementary Table 17: Results of statistical analyses performed for transfections with U6 array and CHIKV luciferase in AF05 cells.

AF05 U6 array

Differences were based on log2 transformed data.

lme model	Sum Sq	Mean Sq	NumDF	DenDF	F value	Pr(>F)
Presence of Cas13b	39.207	39.207	1	134	326.31	<2.20E-16
Position in array	139.363	46.454	3	134	386.63	<2.20E-16
Interaction Cas13:Position	52.132	17.377	3	134	144.63	<2.20E-16

	Estimate	Std. Error	df	t value	Pr(>|t|)
(Intercept)	4.75711	0.08449	46.46171	56.304	< 2e-16
Presence of Cas13b	-0.27864	0.11554	134	-2.412	0.0172
PositionP1	-4.18276	0.11554	134	-36.201	< 2e-16
PositionP2	-2.8161	0.11554	134	-24.373	< 2e-16
PositionP3	-2.26962	0.11554	134	-19.643	< 2e-16
Interaction of Cas13 - PositionP1	3.22253	0.1634	134	19.721	< 2e-16
Interaction of Cas13 - PositionP2	1.39781	0.1634	134	8.554	2.39E-14
Interaction of Cas13 - PositionP3	0.66856	0.1634	134	4.091	7.36E-05

Post-hoc Analysis	Estimate	Std. Error	z value	Pr(>|z|)
Cas13.P1 - Cas13.C	-4.1828	0.1155	-36.201	< 0.001
Cas13.P2 - Cas13.C	-2.8161	0.1155	-24.373	< 0.001
Cas13.P3 - Cas13.C	-2.2696	0.1155	-19.643	< 0.001
ZsG.C - Cas13.C	-0.2786	0.1155	-2.412	0.23553
Cas13.P2 - Cas13.P1	1.3667	0.1155	11.828	< 0.001
Cas13.P3 - Cas13.P1	1.9131	0.1155	16.558	< 0.001
ZsG.P1 - Cas13.P1	2.9439	0.1155	25.479	< 0.001
Cas13.P3 - Cas13.P2	0.5465	0.1155	4.73	< 0.001
ZsG.P2 - Cas13.P2	1.1192	0.1155	9.686	< 0.001
ZsG.P3 - Cas13.P3	0.3899	0.1155	3.375	0.01682
ZsG.P1 - ZsG.C	-0.9602	0.1155	-8.311	< 0.001
ZsG.P2 - ZsG.C	-1.4183	0.1155	-12.275	< 0.001
ZsG.P3 - ZsG.C	-1.6011	0.1155	-13.857	< 0.001
ZsG.P2 - ZsG.P1	-0.4581	0.1155	-3.964	0.00193
ZsG.P3 - ZsG.P1	-0.6408	0.1155	-5.546	< 0.001
ZsG.P3 - ZsG.P2	-0.1828	0.1155	-1.582	0.76168

Random Effects	Variance	Std. Dev.
Experiment	0.00139	0.03729
Residuals	0.12015	0.34663

Supplementary Table 18: Results of statistical analyses performed for transfections with U6 array and CHIKV luciferase in AF319 cells.

AF319 U6 array

Differences based on squareroot transformed data.

lme model	Sum Sq	Mean Sq	NumDF	DenDF	F value	Pr(>F)
Presence of Cas13b	3.328	3.3285	1	125.08	33.779	4.83E-08
Position in array	49.74	16.5801	3	124.99	168.264	< 2.2e-16
Interaction Cas13:Position	11.72	3.9066	3	124.99	39.646	< 2.2e-16

	Estimate	Std. Error	df	t value	Pr(>|t|)
(Intercept)	3.322	0.1787	2.9904	18.588	0.000346
Presence of Cas13b	-0.6287	0.11	125.0315	-5.715	7.62E-08
PositionP1	-2.4891	0.1129	124.9966	-22.055	< 2e-16
PositionP2	-1.8065	0.1129	124.9966	-16.007	< 2e-16
PositionP3	-1.4959	0.1129	124.9966	-13.255	< 2e-16
Interaction of Cas13 - PositionP1	1.6355	0.1539	124.9941	10.627	< 2e-16
Interaction of Cas13 - PositionP2	1.1518	0.1539	124.9941	7.484	1.12E-11
Interaction of Cas13 - PositionP3	0.9937	0.1539	124.9941	6.457	2.14E-09

Post-hoc Analysis	Estimate	Std. Error	z value	Pr(>|z|)
Cas13.P1 - Cas13.C	-2.48907	0.11286	-22.055	<0.001
Cas13.P2 - Cas13.C	-1.80652	0.11286	-16.007	<0.001
Cas13.P3 - Cas13.C	-1.4959	0.11286	-13.255	<0.001
ZsG.C - Cas13.C	-0.62874	0.11002	-5.715	<0.001
Cas13.P2 - Cas13.P1	0.68255	0.11098	6.15	<0.001
Cas13.P3 - Cas13.P1	0.99317	0.11098	8.949	<0.001
ZsG.P1 - Cas13.P1	1.00673	0.10797	9.324	<0.001
Cas13.P3 - Cas13.P2	0.31062	0.11098	2.799	0.0951
ZsG.P2 - Cas13.P2	0.52309	0.10797	4.845	<0.001
ZsG.P3 - Cas13.P3	0.365	0.10797	3.381	0.0163
ZsG.P1 - ZsG.C	-0.85359	0.10463	-8.158	<0.001
ZsG.P2 - ZsG.C	-0.65469	0.10463	-6.257	<0.001
ZsG.P3 - ZsG.C	-0.50215	0.10463	-4.799	<0.001
ZsG.P2 - ZsG.P1	0.1989	0.10463	1.901	0.5495
ZsG.P3 - ZsG.P1	0.35144	0.10463	3.359	0.0181
ZsG.P3 - ZsG.P2	0.15254	0.10463	1.458	0.8297

Random Effects	Variance	Std. Dev.
Experiment	0.07593	0.2756
Residuals	0.09854	0.3139

Supplementary Table 19: Results of statistical analyses performed for transfections with U6 array and CHIKV luciferase in C6/36 cells.

C6/36	U6 array				
Differences based on untransformed data.					
**2-way ANOVA**	**Df**	**Sum Sq**	**Mean Sq**	**F value**	**Pr(>F)**
Presence of Cas13b	1	0.1776	0.17756	28.78	6.32E-05
Position in array	3	0.704	0.23467	38.04	1.65E-07
Interaction Cas13b:Position	3	0.2396	0.07986	12.95	0.000151
Residuals	16	0.0987	0.00617		

Post-hoc Analysis	Estimate	Std. Error	t value	Pr(>|t|)
Cas13.P1 - Cas13.C	-0.658091	0.064131	-10.262	< 0.001
Cas13.P2 - Cas13.C	-0.623092	0.064131	-9.716	< 0.001
Cas13.P3 - Cas13.C	-0.574432	0.064131	-8.957	< 0.001
ZsG.C - Cas13.C	-0.16717	0.064131	-2.607	0.22216
Cas13.P2 - Cas13.P1	0.034999	0.064131	0.546	0.9991
Cas13.P3 - Cas13.P1	0.083659	0.064131	1.305	0.88432
ZsG.P1 - Cas13.P1	0.348772	0.064131	5.438	0.00109
Cas13.P3 - Cas13.P2	0.04866	0.064131	0.759	0.99315
ZsG.P2 - Cas13.P2	0.242615	0.064131	3.783	0.02706
ZsG.P3 - Cas13.P3	0.2639	0.064131	4.115	0.01419
ZsG.P1 - ZsG.C	-0.14215	0.064131	-2.217	0.39185
ZsG.P2 - ZsG.C	-0.213307	0.064131	-3.326	0.06415
ZsG.P3 - ZsG.C	-0.143362	0.064131	-2.235	0.382
ZsG.P2 - ZsG.P1	-0.071157	0.064131	-1.11	0.94539
ZsG.P3 - ZsG.P1	-0.001212	0.064131	-0.019	1
ZsG.P3 - ZsG.P2	0.069945	0.064131	1.091	0.94985

Supplementary Table 20: Primer sequences used in this study. Bold letters: variable guide sequence, underlined letters: T7 promoter.

Segment	For	Primer	Sequence
U6-3 Pol III promoter	AGG1399	2704	ctcattaggcacccaaacacgggagtctggtaaccctagtg
		2705	agcagatagatgagacacatggtctctcaatcgaagagcaacgca
Non variable RNA guide backbone	AGG1399	2706	atgtgtctcatctatctgctggtctcagttgtggaaggtccagttttgaggggct
		2707	atttttaacaaaagctctgtgtcaaaaaaagttgtaatagcccctcaaaactggac
Guide 1	U6-guide 1	2712	attg**gaagaagccgcactgcttcgggtcaccaca**
		2713	caac**tgtggtgacccgaagcagtgcggcttcttc**
Guide 2	U6-guide 2<br>U6-P1<br>U6-P2<br>U6-P3	2714	attg**gtactcattcgtagtgcgcattttgccttc**
		2715	caac**gaaggcaaaatgcgcactacgaatgagtac**
AmC1	U6-P1<br>U6-P2<br>U6-P3<br>U6-PC	2716	attg**ggcaccacggaggtgatgtgggccacggcg**
		2717	caac**cgccgtggcccacatcacctccgtggtgcc**
AmC2	U6-PC	2718	attg**tcccagccggtggtcttcttggccatcacg**
		2719	caac**cgtgatggccaagaagaccaccggctggga**
AmC3	U6-P1<br>U6-P2<br>U6-P3<br>U6-PC	2720	attg**gttcacgccgtggaaggtggacttgtgctc**
		2721	caac**gagcacaagtccaccttccacggcgtgaac**
Template guide 1	*In vitro* transcribed guide 1	2430	gaaattaatacgactcactatag**gaagaagccgcactgcttcgggtcaccaca**gttgtggaaggtccagtt
		2323	gttgtaatagcccctcaaaactggaccttccacaac
Template guide 2	*In vitro* transcribed guide 2	2433	gaaattaatacgactcactatag**gtactcattcgtagtgcgcattttgccttc**gttgtggaaggtccagtt
		2323	gttgtaatagcccctcaaaactggaccttccacaac
Template AmC3	*In vitro* transcribed AmC3	2946	gaaattaatacgactcactatagg**gttcacgccgtggaaggtggacttgtgctc**gttgtggaaggtccagtt
		2323	gttgtaatagcccctcaaaactggaccttccacaac

References

1. Zuker, M. Mfold web server for nucleic acid folding and hybridization prediction. *Nucleic Acids Res.* **31**, 3406–15 (2003).
2. Muckstein, U. *et al.* Thermodynamics of RNA-RNA binding. *Bioinformatics* **22**, 1177–1182 (2006).
